# Clinical and Radiological Evaluation of Flap and Flapless Procedures with Biomaterials in Alveolar Ridge Preservation

**DOI:** 10.3390/jfb16090345

**Published:** 2025-09-14

**Authors:** Ewa Dolińska, Ewa Duraj, Marcin Bernaczyk, Magdalena Sulewska, Małgorzata Pietruska

**Affiliations:** 1 Department of Periodontal and Oral Mucosa Diseases, Medical University of Bialystok, ul. Waszyngtona 13, 15-269 Bialystok, Poland; ewaduraj@gmail.com (E.D.); magdalena.sulewska@umb.edu.pl (M.S.); mpietruska@wp.pl (M.P.); 2 Private Practice, ul. Żeromskiego1A/U1, 15-269 Bialystok, Poland; marcin.bernaczyk@gmail.com; 3 Private Practice, ul. Waszyngtona 1/34, 15-269 Bialystok, Poland

**Keywords:** alveolar ridge preservation, tooth extraction, tooth socket, bone substitutes, biomaterials, bone regeneration

## Abstract

Although ridge preservation procedures have been shown to prevent post-extraction bone loss, the effectiveness of using a flap or flapless surgical approach remains unclear. The aim of the study was to compare the mentioned above alveolar ridge preservation procedures in the esthetic region of maxilla. Twenty-nine patients were randomly assigned to receive flap (*n* = 14) or flapless (*n* = 15) alveolar ridge preservation procedure. Sockets were grafted with alloplastic biomaterial, then covered with a collagen membrane in both groups. Clinical examinations were performed over a 6-month observation period and radiological (CBCT) examination was conducted before and 6 months after treatment. For both after flap and flapless procedures, there was a reduction in interdental papillae height and keratinized tissue width, increase in buccal soft tissues thickness with a decrease in radiological buccal bone plate width, decrease in radiological buccal and lingual plate height (significantly for the flapless group) and radiological alveolar process width reduction (significantly in flapless group at the height of 5 mm and 7 mm from the bottom of the socket). A decrease in the radiological buccal bone plate width was observed, where the further measuring point was from the bottom of the alveolus. In the mucoperiosteal flap preparation, group buccal bone plate width reduction at the height 3 mm, 5 mm and 7 mm was significant and in the flapless group a significant decrease was observed from 5 to 9 mm from the bottom of the socket. Despite ridge preservation, there is soft tissue thickening and a decrease in bone height and width regardless of the flap/flapless method used.

## 1. Introduction

Alveolar bone undergoes processes of apposition and resorption throughout life what allows it to adapt to changing functional requirements [1]. Thickness of the alveolar process affects the course of tissue healing after tooth extraction. The alveolar bone is usually thicker on the palatal/lingual side rather than on the buccal side [2]. Post-extraction bone remodeling occurs both labially and lingually. However, since the vestibular plate is thinner a greater vertical bone loss is observed on the buccal side [3]. During spontaneous healing, a new bone is formed inside the alveolus and there is a reduction in alveolus dimensions. Horizontal bone resorption is significantly greater than vertical [4]. Remodeling of the alveolar process is the most extensive during the first six months after extraction and progresses at an average rate of 0.5–1% per year throughout the course of life [5]. Schropp et al. showed that within 12 months after tooth removal the width of the alveolus decreases by about 50%, with 2/3 of this decrease occurring in the first 3 months after surgery [6].

To reduce changes in the contour of the bone after tooth extraction additional surgical procedures can be performed. With regard to the extension of the surgical field, they can be divided into procedures with or without the mucoperiosteal flap preparation. These surgeries use autogenous bone, biomaterials (xenogeneic, allogeneic, alloplastic), autogenous blood products, biological factors, and different types of barrier membranes or soft tissue grafts. However, no significant differences were noted in the effectiveness of treatment after using the aforementioned biomaterials and different treatment protocols [7,8,9].

Knowledge of the alveolar resorption that occurs after full-thickness flap elevation is well established [10,11]. Flap preparation is believed to cause disruption of the connective tissue attachment to the bone, which at the surface leads to an inflammatory response and resorption of the superficial bone layer in the exposed area [12,13]. Simultaneous tooth removal causes tearing of the blood vessels reaching the alveolar bone resulting in additional necrosis of the superficial bone layer. Since the buccal bone plate is much thinner than the lingual one, this reduction in the horizontal dimension may translate into vertical buccal bone plate resorption [14]. However, a change in alveolar dimensions also occurs after flapless procedures. It is possible that flap elevation affects the change in alveolar dimensions only in the short period of time, while no significant differences are found in the long term. Such conclusions were reached by Araujo and Lindhe [15], who in their study observed that after 6 months the differences between the group with and without flap preparation were imperceptible. The influence of other factors such as bone adaptation to the lack of continuous loading after tooth extraction is also taken into account. In addition, the resorption of the walls of the socket also depends on the loss of the alveolar bone proper (bundle bone), the maintenance of which is conditioned by the presence of the tooth [14]. It has long been assumed that a flapless procedure would be more advantageous than a procedure with flap preparation. However, there is no consensus on this, and various studies present conflicting data.

As few clinical studies undertake a broad range of assessments focusing on single parameters, we decided to conduct a comprehensive clinical assessment of soft tissues and radiological imaging of bone. This included evaluating interdental papillae width and height, keratinized gingiva and soft tissue thickness at three measurement points. For radiological measurements cone beam computed tomography (CBCT) was used as it is the superior method for radiological evaluation and multiplanar image analysis [16,17].

Taking into consideration all above the aim of the study was to compare the effectiveness of two alveolar ridge preservation procedures in the esthetic region of maxilla. The study evaluated treatment with and without the mucoperiosteal flap elevation. To preserve the ridge in both cases the same biomaterials were used. We hypothesized that there are no differences in soft tissue healing or alveolar bone dimensions when using a flap or flapless technique in alveolar ridge preservation procedures. The basis for the comparison was the clinical examination and evaluation of differences in the dimensions of the alveolar process on CBCT scans taken before and 6 months after tooth extraction.

## 2. Materials and Methods

### 2.1. Study Design and Study Groups

The study was planned as a randomized, prospective clinical trial. The project was conducted in accordance with the Helsinki Declaration after obtaining approval from the local bioethics committee (R-I-002/262/2012 and R-I-002/262A/2013).

Generally healthy adults in need of a single tooth extraction in the esthetic zone were eligible. Inclusion criteria were as follows:

- Necessity to remove a single tooth in the maxilla in the esthetic area.
- Minimum age of 18 years.
- Signing a written consent.

Exclusion criteria were as follows.

- Systemic general diseases that affect surgical therapy (uncontrolled diabetes, uncontrolled hypertension, severe heart diseases).
- Osteoporosis treated with bisphosphonates or other antiresorptive drugs.
- Immunosuppression.
- Pregnancy and breastfeeding.
- Smoking.
- Allergy to penicillin.
- Knowledge of allergy to any of the biomaterials used in the study.
- Acute local condition in the area of tooth qualified for removal that could prevent precise performance of the surgical procedure and preparation of temporary work.

**Figure 1:**
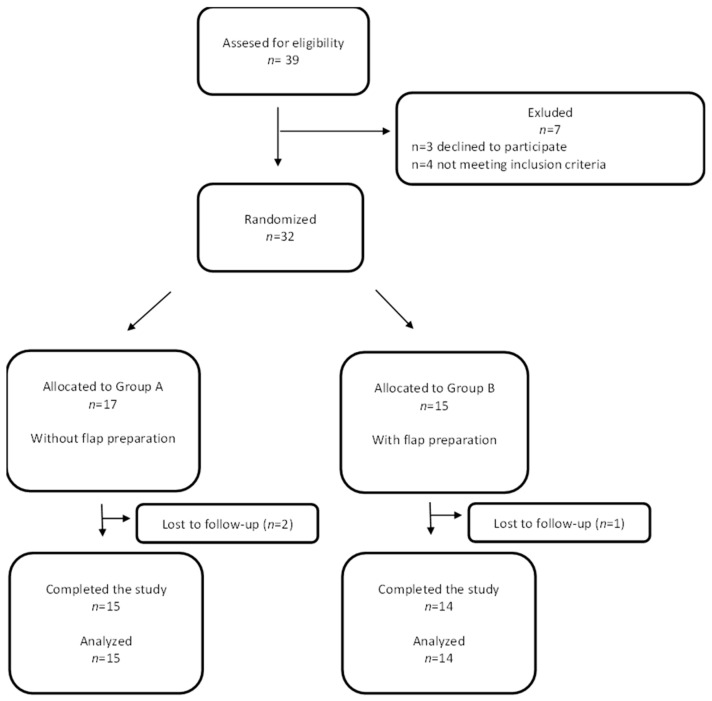
CONSORT flow chart of the study group.

During the qualification period, 39 patients were examined. After initial evaluation, 32 generally healthy adults aged 24–67 years (22 women and 10 men) were qualified for the procedure. Finally, 29 patients aged 24–62 years (19 women and 10 men) who completed a six-month observation period were analyzed. The study started in 2012 and ended in 2019. The process of patient recruitment and randomization, as well as cases excluded from the study, is shown in the Consort flow chart (Figure 1).

Patients were randomized into two study groups based on a randomization computer list. In the first group, tooth extraction was performed without flap elevation (group A, *n* = 15). In the second group, extraction was conducted with mucoperiosteal flap preparation (group B, *n* = 14). The extraction socket was protected with the same biomaterials in both groups.

### 2.2. Clinical and Radiographic Examination

**Figure 2:**
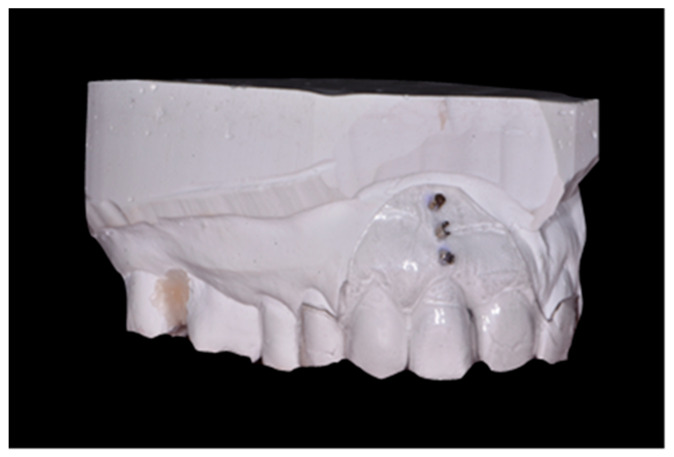
Individual positioner with holes for measuring tool.

Clinical examination was carried out before tooth extraction, 3, 4 and 6 months after surgery. Periodontal examination was performed with a probe PCPUNC 15 (Hu-Friedy, Chicago, IL, USA) calibrated in 1 mm increments. This examination included the following parameters:

- Plaque index (FMPS—full mouth plaque score)—determined dichotomously as the presence or absence of dental plaque on the four tooth surfaces [18].
- Bleeding on probing (FMBOP—full mouth bleeding on probing)—determined dichotomously as bleeding on probing or its absence on 4 tooth surfaces [19].
- Width of keratinized gingiva (KT—keratinized tissue)—measured from the gingival margin to the mucogingival junction in the long axis of the tooth to be removed.
- Papillae height (PHm-mesial, PHd-distal) and papillae width (PWm-mesial, PWd-distal) of the interdental papillae adjacent to the tooth qualified for removal.
- Probing depth (PD), gingival recession (GR), clinical attachment level (CAL) of teeth adjacent to the tooth qualified for extraction.
- Soft tissue thickness in the root projection of the extracted tooth (3 mm, 6 mm and 9 mm from gingival margin of extracted tooth); to measure this parameter, an individual positioner with holes for the measuring tool was prepared before tooth extraction (Figure 2); a sharply pointed root canal tool No. 25 was used for the test; in order to perform measurements in the same point, through the embedded sleeves, a root canal tool No. 25 was passed through and the puncture was performed to determine the measurement point; after removing the stent in the marked areas, the tissue was punctured until palpable bone resistance was felt; measurements were made under local anesthesia.

Radiological examination consisted of dental cone-beam computed tomography (CBCT) performed before and 6 months after tooth extraction. It was used to assess volumetric changes in alveolar process.

### 2.3. Surgical Procedure and Temporary Prosthetic Device

Prior to the extraction, impressions were taken to prepare the temporary prosthetic restoration and to make an individual positioner to measure soft tissue thickness.

Under local anesthesia (40 mg of articaine and 0.012 mg of epinephrine), the qualified tooth was removed. Great care was taken to preserve the alveolar walls.

**Figure 3:**
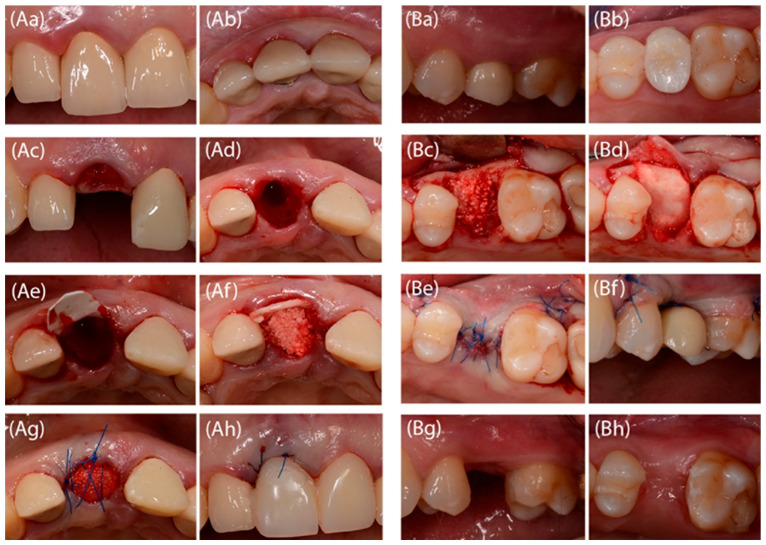
(**Aa**–**Ah**) an example of surgical procedure of tooth extraction with flapless alveolar ridge preservation (Aa-tooth 11 qualified to removal; Ab-occlusal view of 11 tooth; Ac-alveolus after gentle extraction of 11 tooth; Ad-alveolus occlusal view; Ae-status after cone shaped collagen membrane fitting; Af-status after filling the alveolus with alloplastic biomaterial; Ag-non-resorbable suteres; Ah-temporary prosthetic bridge). (**Ba**–**Bh**) an example of surgical procedure of tooth extraction with mucoperiosteal flap preparation (Ba-tooth 25 qualified for extraction; Bb-tooth 25 occlusal view; Bc-status after tooth extraction, mucoperiosteal flap preparation and filling the alveolus with alloplastic biomaterial; Bd-status after collagen membrane fixation; Be-sutures, full wound closure; Bf-temporary prosthetic restoration; Bg-status after 6 months; Bh-status after 6 months, occlusal view).

In patients randomized to group A (without flap preparation), the collagen membrane with extended resorption time (BioMend Extend, Zimmer Dental Inc., Carlsbad, CA, USA) was cut to the shape of the alveolus (an “ice-cream cone” shape). The membrane prepared in this way was placed in the alveolus from the lingual side. The alveolus was then filled with a cone-shaped synthetic biomaterial (99% resorbable β tricalcium phosphate (β-TCP)) (R.T.R., Saint Maur des Fosses, Septodont, France), that was gently condensed to avoid free spaces. The alveolus entrance was closed with a portion of the collagen membrane left outside the alveolus. The wound was secured with knot sutures and a cross-mattress suture (synthetic, non-resorbable, monofilament, polyamide 5.0 surgical threads) to support the membrane (Figure 3A).

In patients randomized to group B a mucoperiosteal flap was prepared. The collagen membrane with extended resorption time (BioMend Extend, Zimmer Dental Inc., Carlsbad, CA, USA) was cut to the shape of the post-extraction site. The membrane was placed under the flap so that it protected the alveolus on each side. The alveolus was then filled with a cone-shaped alloplastic biomaterial (R.T.R., Saint Maur des Fosses, Septodont, France). The biomaterial was gently condensed to avoid free spaces. The alveolar entrance was closed with a collagen membrane and a mucoperiosteal flap was repositioned to ensure a complete wound closure. Analogous sutures were placed as in group A (Figure 3B).

The extraction site was then secured with pre-made temporary prosthetic work. Postoperatively, the patients were prescribed an antibiotic-amoxicillin 1g (Ospamox, Sandoz GmbH, Kundl, Austria), which was taken every 12 h for 7 days. The sutures were removed after 2 weeks.

### 2.4. CBCT Analysis

Cone-beam computed tomographies were performed using a Planmeca Promax 3DS tomograph (Planmeca OY, Helsinki, Finland). The study parameters were as follows: image size: 251 × 251 × 201, voxel size: 200, 90 kV, 12.0 mA, DAP (mGy × cm^2^): 849.

**Figure 4:**
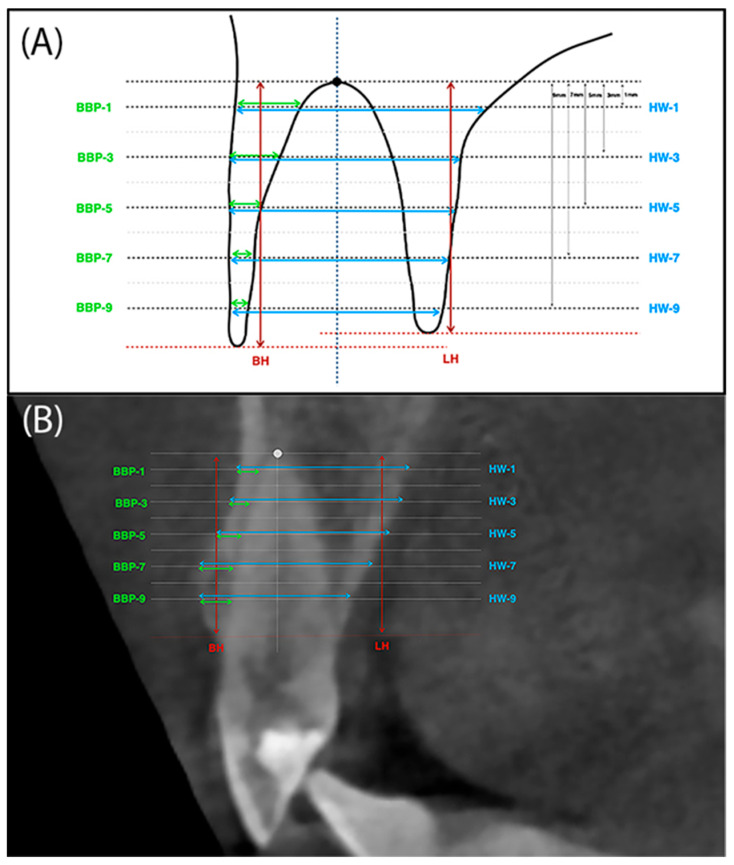
(**A**) Schematic illustration of an alveolus with reference lines drawn. (**B**) Reference lines and points superimposed on an example CBCT scan.

To take measurements on CBCT images, a pre-surgery scan and a follow-up scan-taken after 6 months-were performed. To superimpose the CBCT images, the original Slicer 3.6. software (www.slicer.org) was used. The DICOM (Digital Imaging and Communications in Medicine) files of the two examinations were superimposed with the program by using areas in the images where no changes had occurred in 6 months. Once superimposed, the images were aligned and manually checked for a perfect match. Then, on selected sections of the initial and control study, measurements were taken using the points and reference lines. To determine the points and reference lines, the most apical point of the alveolus was first marked. The vertical line was drawn in the long axis of the tooth through the center of the alveolus, while the horizontal line was drawn so that it was perpendicular to the vertical line and crossed it at the apical reference point (Figure 4A,B). The following sections were measured:

- BH (buccal height)—The height of the buccal plate measured from the edge of the bone margin on the buccal side to the perpendicular line running through the reference point at the alveolar floor.
- LH (lingual height)—The height of the palatal plate measured from the edge of the alveolar bone margin on the buccal side to the perpendicular line running through the reference point at the alveolar floor.
- BBP-1 (buccal bone plate 1)—Thickness of buccal plate measured parallel 1mm from the reference line running through the alveolar floor and analogously BBP-3, BBP-5, BBP-7 and BBP-9.
- HW-1 (horizontal width 1)—The width of the alveolus measured parallel 1mm from the reference line running through the bottom of the alveolus. And analogously HW-3, HW-5, HW-7 and HW-9 (Figure 4A,B).

### 2.5. Statistical Analysis

Variables were featured by descriptive statistics parameters (i.e., arithmetic mean, median and standard deviation). Normality distribution was checked using the Shapiro–Wilk test. For clinical data, ANOVA, MANOVA or Friedman’s test was used for comparisons over time, along with Dunn’s multiple comparisons test with Bonferroni correction. Student’s *t*-test, Cochran-Cox or Mann–Whitney test was used for comparisons of parameters between groups. In the analysis of radiological data, Student’s *t*-test or Wilcoxon’s test was used for comparisons of study parameters over time, and Student’s *t*-test or Mann–Whitney test was used for comparisons of parameters between groups (depending on whether the assumption of normality of distribution was met). Results were considered statistically significant at *p* < 0.05. PQStat software version 1.8.0.476 was used for analysis.

## 3. Results

Finally, 29 patients were analyzed (15 in group without flap preparation; 14 in group B, group with flap formation). Demographic characteristics of the study groups and the teeth’s positions and causes of tooth loss are shown in Table 1. None of the patients reported adverse events.

### 3.1. Clinical Examination

Changes in FMPS and FMBOP indices in both groups at 6-month follow-up are shown in Supplementary Materials (Table S1) as well as the periodontal parameters of the teeth adjacent to the tooth extracted/gap on the mesial (m) and distal (d) sides such as PD, GR and CAL (Table S2). No differences were noted between the groups according to these parameters.

A statistically significant reduction in mesial and distal interdental papillae height was observed after tooth extraction in both study groups (Table 2).

Keratinized tissue width (Table 3) showed a statistically significant decrease in the flapless group from 6.5 mm to 5.4 mm and from 4.79 mm to 3.82 mm in the group with flap preparation at six-month follow-up. Groups A and B differed with respect to this parameter before extraction and after 3, 4 and 6 months. After 3 months, the mean difference in KT was 0.97 mm in group A and 1.18 mm in group B. After 6 months, these differences were reversed, and so in group A, KT decreased by 1.1 mm on average and in group B by 0.96 mm.

Soft tissue thickness measured at points determined by the individually prepared positioner at a height of 3 mm from the gingival margin increased significantly in both groups, and this was the increase noted between the entrance examination and each subsequent time point. At a height of 6 mm there was also a statistical increase in soft tissue thickness. The measurement at 9 mm was not significant in the flapless group. In the group with flap preparation, an increase in soft tissue thickness was observed and post hoc tests showed significance between the basic and 3 months examination. Differences between groups A and B were noted only at 9 mm at 3 and 4 months after surgery. Greater soft tissue thickness was described in the group with flap preparation (Table 4).

### 3.2. Radiological Examination-Evaluation of CBCT Scans

**Figure 5:**
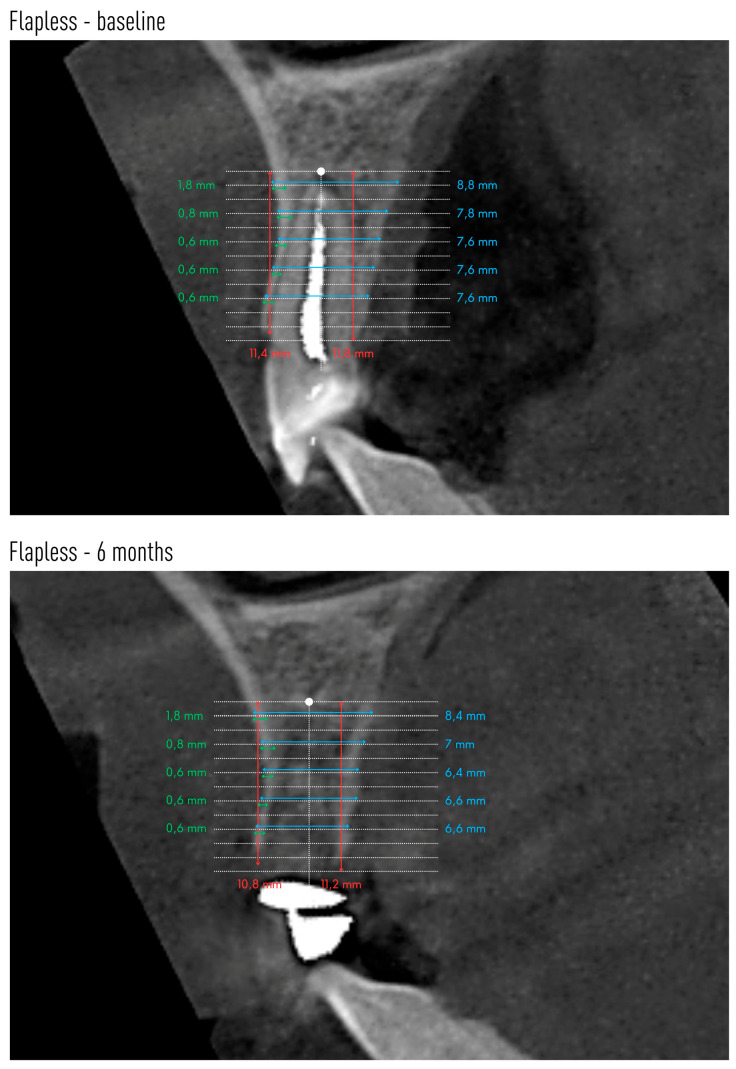
An example CBCT scans of a patient allocated to flapless group with reference lines and measurements made on baseline and after 6 months.

**Figure 6:**
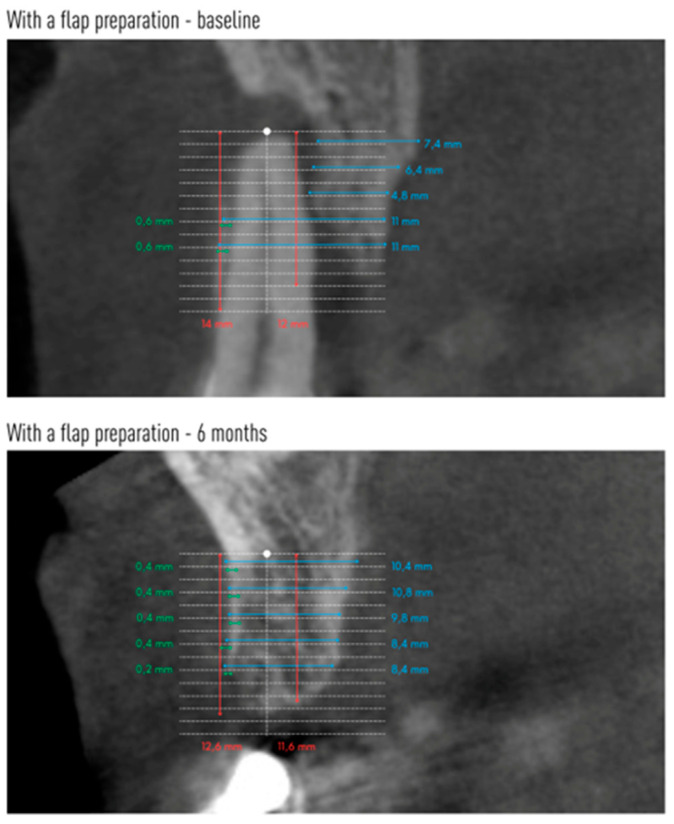
An example CBCT scans of a patient allocated to flap group with reference lines and measurements made on baseline and after 6 months.

Cone-beam computed tomography (CBCT) was performed before tooth extraction and 6 months after the procedure. Sample scans with measurements marked are shown in Figure 5 (flapless group) and Figure 6 (flap group).

Changes in the mean height of the buccal (BH) and lingual (LH) alveolar bone plates are shown in Table 5. The height of both plates decreased in both groups after 6 months. However, significant radiographic changes in BH and LH were observed only in the group without preparation of the mucoperiosteal flap. In group A, the buccal plate was reduced by an average of 1.08 mm, and the lingual plate by 1.29 mm. There were no statistical differences between the groups.

Changes in the anteroposterior alveolar buccal bone plate thickness (BBP) at 1 mm, 3 mm, 5 mm, 7 mm and 9 mm from the alveolar floor are shown in Table 6. The two groups did not differ with respect to these radiographic parameters before tooth extraction or 6 months after (except for BBP-9 before extraction). However, a decrease in the radiographic width of the vestibular lamina was observed, the greater the farther the measuring point was located from the alveolar floor. In the group with flap preparation, already at a height of 3 mm from the alveolar bottom, as well as 5 mm and 7 mm from the alveolar floor, the reduction in the width of the bone gained statistical significance. At a height of 9 mm from the alveolar floor, the average thickness of BBP9 was only 0.01 mm. In the flapless group, a significant decrease in the thickness of the vestibular compact plate was observed from BBP5 to BBP9.

Table 7 shows the changes in the mean alveolar horizontal width of the entire alveolar process. The radiographic width of the alveolar process was smaller 6 months after tooth extraction at each height in both groups. However, this reduction reached statistical significance only at heights of 5 mm and 7 mm in the flapless group.

## 4. Discussion

The purpose of this study was to evaluate the maxillary alveolar process changes after tooth extraction in an alveolar preservation procedure (ARP) using collagen membrane and alloplastic biomaterial performed with or without a mucoperiosteal flap preparation.

Eligible patients were characterized by good oral hygiene and little bleeding on probing (low FMPS and FMBOP). Probing depths (PD) at the teeth adjacent to the removed one did not change, gingival recessions (GR) did not deepen. There was also no significant clinical attachment level loss (CAL) over time in either group. Over the course of the study, patients were supplied with temporary prosthetic restoration, which was designed to shape soft tissues and maintain papillae height. Despite this, there was a reduction in the average height of the papillae mesially and distally in both groups, and these were significant changes in height over time. In the flap preparation group, the mean mesial papillae height was significantly lower than after the flapless procedure at 6 months. There was also a reduction in the average papillae width over time in both groups. The reduction in the width of the distal papilla was significant over time. These results are as expected because during flap preparation the treatment area is widened, the papillae are debrided and then secured with sutures. The formation of recessions at the adjacent teeth and damage to the interdental papillae have also been described by other authors, who associated these adverse changes with manipulations during preparation of the mucoperiosteal flap [20]. The reduction in the height of the interdental papillae in the group without flap preparation is also not surprising because the absence of a tooth results in alveolar remodeling and reduced bony support for soft tissues.

In both groups, the width of the attached gingiva after tooth extraction decreased significantly. At each stage of the study, the mean KT value was higher in the group in which the flap was not prepared. As the groups differed in terms of KT before tooth extraction, the mean differences in the two groups were compared over time. Thus, after 3 months, KT decreased by 0.97 mm in group A and by 1.18 mm in group B. This indicates a short-term advantage of the procedure without flap preparation. The benefit of flapless preparation, however, was not apparent after 6 months because at this time point the loss of KT in the flapless group was 1.1 mm and in the group with flap preparation 0.96 mm. However, it can be concluded that the differences between the groups were small. Positive changes in the width of the keratinized tissue after procedures without flap preparation have been observed by many authors. Hong et al., obtained a 0.43 mm increase in KT width after flapless procedures, compared to a loss of 1.57 mm in the group with flap preparation [21]. Similarly, Barone et al., obtained a 1.8 mm increase in KT width in the group without flap preparation and a 1.7 mm loss in the procedure with flap preparation [22]. Convergent observations were also made by other authors, who found preservation of greater KT width in procedures without flap preparation [12,23]. Such large differences between the groups are not confirmed by our observations.

Measurements of soft tissue thickness on the labial side in the axis of the extracted tooth were taken at three measurement points determined by the custom-made positioner (3 mm, 6 mm and 9 mm from the gingival margin of the tooth qualified for extraction). At 3 mm from the gingival margin, that is, at the measurement point closest to the alveolar margin, a large increase in soft tissue thickness was noted. In both groups, this was a statistically significant increase between the pre-extraction examination and each subsequent time point. At a height of 6 mm, an increase in soft tissue thickness was also noted in both groups, but in the group without flap preparation it was on the borderline of significance (*p* = 0.0488). At a height of 9 mm, labial soft tissue thickness was higher only in the group with flap preparation, and this was a small increase of 0.39 mm at 6 months. At a height of 9 mm at three-month and four-month follow-up, the groups differed, with greater soft tissue thickness recorded in the group where the flap was prepared. This indicates that there was significant remodeling of the alveolar process after tooth extraction in the midline and a partial replacement of hard tissues by healed soft tissues. In flap preparation group, this remodeling is seen farther from the alveolar ridge compared to the flapless procedure. An increase in soft tissue thickness on the labial side after tooth extraction with an alveolar preservation procedure has also been obtained by other researchers [24]. Volumetric evaluation of soft tissues on models made before extraction and 6 months after was attempted by Schneider et al. [17]. The models made were scanned and volumetric changes in the soft tissue on the vestibular side were measured digitally. They found changes in the width of the soft tissues on the labial side regardless of the use or not of procedures preserving the ridge [17].

Performing CBCT allowed measurements of alveolar process bone height and width. Measurements of the height of the labial plate (BH) showed its significant reduction after 6 months in the group without flap preparation. The difference was 1.08 mm or 13.2%. In the group with a full-thickness flap, the reduction in the height of the labial plate also occurred but to a much lesser extent (0.49 mm, or 6.1%), and these changes were not significant. The change in the height of the alveolar lingual plate was similar. In group A, the change in height was significant at 1.29 mm (14.8%) after 6 months. In group B, there was also a reduction in the height of the lingual plate by an average of 0.46 mm, or 5.2% (not significant). There were no statistical differences between the groups. A similar model for radiographic examination of the post-extraction alveolus was used by Jung et al., where the height of the labial and lingual lamina on CBCT scans was evaluated in 4 study groups [25]. Spontaneous healing of the alveolus resulted in 0.5 mm loss of the labial plate and 0.6 mm loss of the lingual plate of the alveolus. Filling the alveolus with β-TCP resulted in a loss of as much as 2 mm of labial plaque and 1.7 mm of lingual plaque. After xenograft and collagen matrix, there was no change in the height of the labial plate, and the lingual plate decreased by 0.4 mm. Socket augmentation with xenograft and preservation of the alveolus entrance with autologous graft was the most favorable and resulted in a 1.2 mm gain in the labial plate and 0.3 mm in the lingual plate of the alveolus [25]. Even better results of augmentation of severely resorbed alveoli after using collagen membrane and xenogeneic material were described by Manavella et al. [26]. One year after the procedures, they found a loss of only 0.31 mm of lingual lamina and a gain of 2.08 mm of alveolar labial lamina height.

The CBCT scans also enabled assessment of the vestibular buccal bone plate thickness at five measurement points. The smallest changes in the width of the labial plate were seen in the most apical part of the socket. Thus, at a height of 1 mm from the reference point at the bottom of the alveolus, they were almost imperceptible. At a height of 3 mm in the group with flap preparation, the thickness of the labial lamina decreased by 0.27 mm. At a height of 5 mm, the reduction in the thickness of the vestibular bone plate was evident in both groups and was 0.24 mm in the flapless procedure and 0.36 mm in the flap preparation group, respectively. At a height of 7 mm, the differences in atrial plate thickness were even more marked, amounting to 0.37 mm in group A and 0.46 mm in group B. The farther the reference point was from the alveolar floor, the greater the loss of vestibular plate thickness occurred. At a height of 9 mm in the flap preparation group, the thickness of the vestibular plate 6 months after extraction was only 0.01 mm. However, evaluating the scans after 6 months, there were no differences between the groups at any measurement point, although the averaged data indicate a greater loss of alveolar vestibular bone thickness in the group after mucoperiosteal flap preparation.

The entire alveolar process thickness (HW) was also assessed at the same heights as the BBP. Alveolar process width was already smaller at heights of 1 mm and 3 mm from the alveolus bottom in both groups, but these values were not significant. At a height of 5 mm and 7 mm, the width of the alveolus significantly decreased in the group without flap preparation by 0.79 mm and 1.12 mm, respectively. The width of the alveolar process also decreased in the group with flap preparation but not significantly. Differences in the width of the alveolus before and after extraction were higher for group A.

Flap and flapless treatment options have been compared in animal studies and clinical trials. In a study on dogs, Fickl et al., showed that tooth removal after flap elevation allowing the alveolar bone margin to become visible resulted in a 14% reduction in hard and soft tissues compared to the flapless procedure [27]. Similar results in a study on dogs were obtained by Blanco et al. [28]. After implantation into the sites where ridge was preserved with and without flap preparation, after 3 months they obtained about 0.5 mm difference in the height of the labial plate in favor of the flap “free” procedure (without significance). In another study by the same group evaluating changes in alveolar height and width, the results of surgeries with and without flap were similar [29]. However, the follow-up in this study was only 3 months. Araujo et al., did not confirm the superiority of flapless treatments. In a study on dogs, they found changes of about 35% reduction in hard tissue in both groups [15].

Clinical studies have also been inconclusive. Barone et al. valuated a procedure with flap preparation and complete post-extraction wound closure and a flapless procedure [22]. Three months after filling the alveolus with xenograft and closing with a collagen membrane, they examined the amount of newly formed bone, residual biomaterial and the presence of marrow spaces. The researchers found no histological or histomorphometric differences between the groups [22]. In another study by the same group, more adverse changes were noted after flap preparation, including a greater loss of alveolar process width. More KT was observed without flap preparation, but in this case the height of the labial plate was lower than with the procedure with flap preparation [30]. Preservation of a wider zone of keratinized tissue and less postoperative discomfort and swelling after procedures without flap preparation was reported by Engler-Hamm et al. [23]. However, 27% to 30% loss of alveolar process width was reported after both types of procedures [23]. Subsequent randomized clinical trials were also inconclusive and did not confirm the superiority of either procedure [31]. This is also confirmed by the results of our study, in which both procedures yield similar clinical and radiological results. It can be assumed that tooth extraction causes alveolar trauma (soft tissues are torn, periodontal vessels are damaged or destroyed), which overlaps with trauma after flap elevation. This somewhat offsets the sheer effect of flap preparation on alveolar resorption [3].

A 2012 systematic review summarized the benefits of alveolar preservation procedures, namely significantly less vertical and horizontal alveolar ridge resorption. The advantages of the procedure with flap elevation, including alveolar ridge width preservation, were also noted. More favorable healing in the procedure with elevated flap may be related to complete postoperative wound closure, including regenerative membrane and primary intention healing [32]. Better outcomes after mucoperiosteal flap preparation were also confirmed by another systematic review from 2014 [33]. The lack of clear recommendations on how to perform alveolar-preserving procedures is also apparent from recent systematic reviews [34,35]. The 2022 analysis concluded that changes in alveolar height and width after both types of procedures were comparable. However, thicker soft tissues on the labial side and more favorable changes regarding keratinized tissue width, as well as less post-operative pain were observed with the flapless approach [35].

Another aspect of alveolar ridge preservation procedures is the use of biomaterials. In our study, it was the collagen membrane with extended resorption time BioMend Extend (Zimmer Dental Inc., Carlsbad, CA, USA) and the alloplastic biomaterial composed of β tricalcium phosphate (β-TCP) and collagen R.T.R. cone (Septodont, Saint-Maur-des-Fosses, France). As the membrane is exposed to the oral environment in the flapless procedure, we opted for a collagen membrane with an extended resorption time. We chose for a β-tricalcium phosphate preparation as the alveolar socket filler due to its high resorbability, which allows for a small amount of biomaterial to remain in the bone-like tissue that fills the alveolus after healing. In vitro studies have shown that highly cross-linked membranes are more resistant to lysis caused by bacterial proteases [36], which may have implications for their longer duration when healing without full flap closure. Numerous papers, including a 2013 systematic review, confirm the benefits of using a collagen membrane [37]. It can be speculated that the membrane, which has a protective effect on the wound and maintains space for the clot, enhances the physiologic healing process by minimizing bone loss [37]. The use of xenogeneic or allogeneic material with a collagen membrane [38,39] or the use of biomaterial and collagen membrane in a technique with full-thickness flap preparation [33] was particularly beneficial for maintaining the width of the alveolar process, which is also confirmed by our results. Recent systematic review confirmed that usage of collagen membrane and bone graft is advantageous for preserving alveolar width and height [40] and the other concluded that clinicians should choose membranes for sealing tooth sockets over biomaterial fillers because this minimizes post-extraction resorption [41]. The healing period is also important because, over time, the alveolar ridge becomes increasingly mineralized and contains fewer and fewer particles of biomaterial [42].

When conducting an alveolar-preserving procedure with flap preparation, there is the possibility of complete membrane closure. This can favorably affect the outcome. Flapless procedures do not offer this possibility and the part of the membrane exposed to the oral cavity environment can easily become contaminated. In guided tissue regeneration (GTR), membrane exposure is considered a complication and is associated with poorer outcomes of reconstruction [43]. In the case of guided bone regeneration (GBR), earlier work suggested that membrane exposure may also translate into a worse outcome [44,45]. However, more recent publications show that intentional exposure of the resorbable membrane does not adversely affect the results of GBR [46].

Despite our extensive analysis of clinical and radiological data, our work is not without limitations. One is the inclusion of only non-molar sites in the maxilla. Therefore, the results of the study should be extrapolated with caution to molars and mandibular teeth. Furthermore, the study focused on incisors, canines and premolars, which may introduce bias due to possible differences in bone remodeling patterns between tooth groups. Histological evaluation would be as well beneficial. However, as most of the patients studied opted for traditional fixed restoration treatment, sampling of bone specimens would have been unethical. Further research is therefore needed to confirm our findings and establish the most effective treatment for alveolar ridge preservation procedure. Recent systematic reviews [38] also highlight the need for new randomized clinical trials that focus specifically on direct comparisons and the long-term evaluation of the ridge preservation procedures These trials should also verify whether the flap or flapless technique significantly affects the outcome of implant placement and the red esthetics around implant crowns. Assessing patient-related outcomes is also of interest.

## 5. Conclusions

Both after the alveolar ridge preservation procedure with and without flap preparation, the following occurs:

- A reduction in interdental papillae height and keratinized tissue width.
- Increase in buccal soft tissues thickness at a height of 3 mm and 6 mm from the gingival margin.
- Decrease in radiological buccal bone plate width.
- Decrease in radiological buccal and lingual plate height (significantly for the group without flap preparation).
- Radiological alveolar process width reduction.

Based on the study, it is not possible to determine the superiority of any of the procedures studied.

## Figures and Tables

**Table 1:** Demographic characteristics of the study groups and the reasons for tooth loss and positions of the teeth qualified for removal.

	Flapless (Group A)	With a Flap (Group B)
*n*	15	14
Female/Male	10F, 5M	9F, 5M
Mean age ± SD	43 ± 10 years	43 ± 10 years
Age range	27–62 years	24–59 years
Tooth Position:		
incisor	12	5
canine	0	0
premolar	3	9
Reasons for Tooth Loss:		
fracture/rupture of a tooth	9	4
endodontic treatment failure	5	7
root resorption	1	2
periodontitis	0	1
other	0	0

**Table 2:** Changes in the height and width of the mesial and distal papillae of a tooth scheduled for removal (PH—papilla height, PW—papilla width, m—mesial, d—distal).

		Baseline (0)	3 Months	4 Months	6 Months	*p*-Time Changes
**PHm**	Flapless group (Group A) Flap group (Group B) p* between groups	3.37 ± 1.04 ^ 2.75 ± 1.05 p* = ns	2.50 ± 0.94 ^ 2.11 ± 0.76 p* = ns	2.80 ± 0.96 2.14 ± 0.86 p* = ns	3.10 ± 1.23 2.21 ± 0.73 p* = 0.028	*p* = 0.0011 *p* = 0.0068
**PWm**	Flapless group (Group A) Flap group (Group B) p* between groups	4.93 ± 0.80 4.79 ± 0.80 p* = ns	4.80 ± 0.77 4.50 ± 0.94 p* = ns	4.73 ± 0.80 4.50 ± 0.85 p* = ns	4.73 ± 0.59 4.21 ± 0.97 p* = ns	*p* = ns *p* = ns
**PHd**	Flapless group (Group A) Flap group (Group B) p* between groups	2.97 ± 0.97 ^ 3.07 ± 1.22 ^ p* = ns	2.17 ± 0.92 ^ 2.18 ± 1.27 p* = ns	2.47 ± 1.20 2.04 ± 1.06 p* = ns	2.67 ± 1.36 1.86 ± 1.08 ^ p* = ns	*p* = 0.0043 *p* = 0.0022
**PWd**	Flapless group (Group A) Flap group (Group B) p* between groups	5.37 ± 0.90 5.46 ± 1.69 p* = ns	4.67 ± 0.72 4.43 ± 1.22 p* = ns	4.40 ± 0.91 4.36 ± 1.08 p* = ns	4.53 ± 0.74 4.43 ± 1.09 p* = ns	*p* < 0,0001 *p* = 0.0122

^ sign denote significance between time points as shown by Dunn’s post hoc tests with Bonferroni correction, ns—non significant, p*—between groups.

**Table 3:** Mean keratinized tissue (KT) width in each group at six-month follow-up with mean differences over time.

		Baseline (0)	3 Months	4 Months	6 Months	*p*	Diff ± SD (0–3m)	Diff ± SD (0–4m)	Diff ± SD (0–6m)
**KT**	Flapless group Flap group p* between groups	6.5 ± 2.54 4.79 ± 2.01 p* = 0.021	5.53 ± 2.36 3.61 ± 1.47 p* = 0.027	5.50 ± 2.29 3.82 ± 1.64 p* = 0.032	5.40 ± 2.10 3.82 ± 1.96 p* = 0.046	*p* = 0.015 *p* = 0.04	0.97 ± 1.3 1.18 ± 1.54	1.0 ± 1.3 0.96 ± 1.55	1.1 ± 1.17 0.96 ± 1.97

p*—between groups.

**Table 4:** Average soft tissue thickness measurements using an individual positioner—3 mm, 6 mm, 9 mm from the gingival margin of the tooth to be removed.

		Baseline (0)	3 Months	4 Months	6 Months	*p*
**Measurement on 3 mm**	Flapless group (Group A) Flap group (Group B) p* between groups	2.07 ± 0.86 2.79 ± 2.46 p* = ns	7.03 ± 2.58 6.43 ± 3.16 p* = ns	7.00 ± 2.33 6.25 ± 3.07 p* = ns	6.60 ± 2.51 6.43 ± 3.01 p* = ns	*p* < 0.0001 *p* = 0.0002
**Measurement on 6 mm**	Flapless group (Group A) Flap group (Group B) p* between groups	2.97 ± 1.38 2.64 ± 1.98 *p* = ns	4.73 ± 2.16 5.36 ± 2.82 *p* = ns	4.27 ± 2.15 4.75 ± 1.90 *p* = ns	3.77 ± 1.66 4.54 ± 1.89 *p* = ns	*p* = 0.0488 *p* = 0.0008
**Measurement on 9 mm**	Flapless group (Group A) Flap group (Group B) p* between groups	3.23 ± 2.51 3.11 ± 2.14 p* = ns	3.37 ± 1.54 5.46 ± 3.75 p* = 0.046	2.87 ± 1.20 4.11 ± 1.30 p* = 0.013	2.67 ± 0.98 3.50 ± 1.51 p* = ns	*p* = ns *p* = 0.007

ns—non significant, p*—between groups.

**Table 5:** Mean changes in buccal (BH) and lingual (LH) alveolar bone plates height assessed on CBCT scans before and 6 months after tooth extraction.

		Baseline (0)	6 Months	*p*	Diff ± SD
**BH**	Flapless group (Group A) Flap group (Group B) p* between groups	8.19 ± 3.74 7.99 ± 3.93 p* = ns	7.11 ± 2.85 7.50 ± 2.40 p* = ns	*p* = 0.0353 *p* = ns	−1.08 ± 1.90 −0.49 ± 3.45
**LH**	Flapless group (Group A) Flap group (Group B) p* between groups	8.69 ± 2.98 8.91 ± 2.63 p* = ns	7.40 ± 2.32 8.46 ± 2.09 p* = ns	*p* = 0.0046 *p* = ns	−1.29 ± 1.49 −0.46 ± 1.38

ns—non significant, p*—between groups.

**Table 6:** Mean changes in buccal bone plate (BBP) thickness assessed on CBCT scans before and 6 months after tooth extraction.

		Baseline (0)	6 Months	*p* in Time	Diff ± SD
**BBP-1**	Flapless group (Group A) Flap group (Group B) p* between groups	0.68 ± 0.51 1.03 ± 1.10 p* = ns	0.71 ± 0.47 0.87 ± 1.07 p* = ns	*p* = ns *p* = ns	0.03 ± 0.15 −0.16 ± 0.42
**BBP-3**	Flapless group (Group A) Flap group (Group B) p* between groups	0.51 ± 0.35 0.81 ± 0.86 p* = ns	0.47 ± 0.34 0.54 ± 0.77 p* = ns	*p* = ns *p* = 0.0342	−0.04 ± 0.11 −0.27 ± 0.43
**BBP-5**	Flapless group (Group A) Flap group (Group B) p* between groups	0.57 ± 0.45 0.61 ± 0.63 p* = ns	0.33 ± 0.41 0.26 ± 0.31 p* = ns	*p* = 0.0156 *p* = 0.0161	−0.24 ± 0.33 −0.36 ± 0.55
**BBP-7**	Flapless group (Gorup A) Flap group (Group B) p* between groups	0.63 ± 0.74 0.50 ± 0.63 p* = ns	0.25 ± 0.37 0.04 ± 0.12 p* = ns	*p* = 0.0312 *p* = 0.0039	−0.37 ± 0.62 −0.46 ± 0.62
**BBP-9**	Flapless group (Group A) Flap group (Group B) p* between groups	0.67 ± 0.74 0.19 ± 0.27 p* = 0.0312	0.24 ± 0.38 0.01 ± 0.05 p* = ns	*p* = 0.0078 *p* = ns	−0.43 ± 0.64 −0.17 ± 0.25

ns—non significant, p*—between groups.

**Table 7:** Mean changes in alveolar width (HW) of 1 mm (HW-1), 3 mm (HW-3), 5 mm (HW-5) and 9 mm (HW-9) from the alveolar floor assessed on CBCT scans before and 6 months after tooth extraction.

		Baseline (0)	6 Miesięcy	*p* Time Changes	Diff ± SD
**HW-1**	Flaplesss group (GroupA) Flap group (Group B) p* between groups	7.87 ± 2.37 10.19 ± 2.99 p* = 0.0280	7.29 ± 2.04 9.79 ± 2.29 p* = 0.0016	*p* = ns *p* = ns	−0.57 ± 1.22 −0.40 ± 2.20
**HW-3**	Flapless group (Group A) Flap group (Group B) p* between groups	7.15 ± 2.80 9.36 ± 2.95 p* = 0.0490	6.64 ± 1.69 8.94 ± 2.05 p* = 0.0026	*p* = ns *p* = ns	−0.51 ± 2.05 −0.41 ± 2.72
**HW-5**	Flapless group (Group A) Flap group (Group B) p* between group	6.23 ± 3.93 7.86 ± 3.99 p* = ns	5.44 ± 2.40 7.73 ± 2.79 p* = 0.0251	*p* = 0.0156 *p* = ns	−0.79 ± 2.31 −0.13 ± 3.69
**HW-7**	Flapless group (Group A) Flap group (Group B) p* between groups	5.43 ± 4.44 6.24 ± 4.83 p* = ns	4.31 ± 3.25 5.15 ± 3.14 p* = ns	*p* = 0.0186 *p* = ns	−1.12 ± 1.56 −1.09 ± 3.67
**HW-9**	Flapless group (Group A) Flap group (Group B) p* between groups	3.72 ± 4.44 3.15 ± 4.61 p* = ns	2.25 ± 3.15 2.44 ± 3.05 p* = ns	*p* = ns *p* = ns	−1.47 ± 5.31 −0.71 ± 2.97

ns—non significant, p*—between groups.

## Data Availability

The original contributions presented in the study are included in the article, further inquiries can be directed to the corresponding author.

## Supplementary Materials

The following supporting information can be downloaded at: https://www.mdpi.com/article/10.3390/jfb16090345/s1, Table S1: Plaque (FMPS) and bleeding (FMBOP) indices characterizing the whole oral cavity.; Table S2: Periodontal clinical parameters of the teeth adjacent to the tooth scheduled for extraction (PD—probing depth, GR—gingival recession, CAL—clinical attachment level, m–mesial, d—distal).

## Abbreviations

The following abbreviations are used in this manuscript:

ARP	Alveolar Preservation Procedure
BBP	buccal bone plate
BH	buccal height
CAL	clinical attachment level
CBCT	cone beam computed tomography
FMBOP	full mouth bleeding on probing
FMPS	full mouth plaque score
GR	gingival recession
HW	horizontal width
KT	keratinized tissue
LH	lingual height
PD	probing depth
PH	papilla height
PW	papilla width
