# Supplementary material for: Clinical and Radiological Evaluation of Flap and Flapless Procedures with Biomaterials in Alveolar Ridge Preservation
Source: J Funct Biomater. 2025 Sep 14;16(9):345. doi: 10.3390/jfb16090345 (PMC12470390; doi:10.3390/jfb16090345)
Supplement: Supplementary file 1 [file jfb-16-00345-s001.zip › jfb-3839213-supplementary.pdf]

Table S1. Plaque (FMPS) and bleeding (FMBOP) indices for the whole oral cavity.

	Baseline (0)	3 Months	4 Months	6 Months	*p*
**FMPS**<br>Flapless groups (Group A)<br>Flap group (Gruop B)<br>*p** between groups	27.45 ± 19.61<br>19.76 ± 15.12<br>*p** = ns	28.56 ± 23.61<br>21.70 ± 12.59<br>*p** = ns	26.88 ± 20.43<br>19.40 ± 12.20<br>*p** = ns	26.29 ± 21.11<br>17.59 ± 14.22<br>*p** = ns	*p* = ns<br>*p* = ns
**FMBOP**<br>Flapless group (Group A)<br>Flap group (Group B)<br>*p** between groups	23.24 ± 18.43<br>19.45 ± 11.48<br>*p**= ns	19.17 ± 16.58<br>18.30 ± 10.33 p*<br>*p**= ns	14.60 ± 8.80<br>13.12 ± 7.71<br>*p** = ns	15.52 ± 10.76<br>13.62 ± 9.37<br>*p** = ns	*p* = 0.0072<br>*p* = ns

Arrows denote significance between studies as shown by Dunn's *post hoc* tests with Bonferroni correction.

Table S2. Periodontal clinical parameters of the teeth adjacent to the tooth scheduled for extraction (PD-probing depth, GR-gingival recession, CAL-clinical attachment level, m-mesial, d-distal).

	Baseline(0)	3 Months	4 Months	6 Months	*p*
**PDm**<br>Flapless group (Gruop A)<br>Flap group (Gruop B)<br>*p** between groups	2.99 ± 0.63<br>2.98 ± 0.82<br>*p** = ns	2.74 ± 0.61<br>3.00 ± 0.83<br>*p** = ns	2.75 ± 0.62<br>2.91 ± 0.82<br>*p** = ns	2.75 ± 0.59<br>2.76 ± 0.83<br>*p** = ns	*p* = ns<br>*p* = ns
**GRm**<br>Flapless group (Gruop A)<br>Flap group (Gruop B)<br>*p** between groups	0.21 ± 0.74<br>0.13 ± 0.40<br>*p** = ns	0.21 ± 0.50<br>0.14 ± 0.40<br>*p** = ns	0.22 ± 0.51<br>0.20 ± 0.41<br>*p** = ns	0.21 ± 0.48<br>0.21 ± 0.41<br>*p** = ns	*p* = ns<br>*p* = ns
**CALm**<br>Flapless group (Gruop A)<br>Flap group (Gruop B)<br>*p** between groups	3.20 ± 1.04<br>3.11 ± 0.88<br>*p** = ns	2.94 ± 0.85<br>3.14 ± 0.98<br>*p** = ns	2.97 ± 0.87<br>3.11 ± 0.96<br>*p** = ns	2.96 ± 0.85<br>2.97 ± 1.00<br>*p** = ns	*p* = ns<br>*p* = ns
**PDd**<br>Flapless group (Gruop A)<br>Flap group (Gruop B)<br>*p** between groups	2.78 ± 0.61<br>3.26 ± 0.79<br>*p** = ns	2.55 ± 0.51<br>2.99 ± 0.66<br>*p** = ns	2.60 ± 0.51<br>2.91 ± 0.57<br>*p** = ns	2.55 ± 0.54<br>2.80 ± 0.70<br>*p** = ns	*p* = ns<br>*p* = ns
**GRd**<br>Flapless group (Gruop A)<br>Flap group (Gruop B)<br>*p** between groups	0.07 ± 0.26<br>0.13 ± 0.40<br>*p** = ns	0.12 ± 0.30<br>0.19 ± 0.42<br>*p** = ns	0.13 ± 0.30<br>0.17 ± 0.42<br>*p** = ns	0.12 ± 0.30<br>0.23 ± 0.48<br>*p** = ns	*p* = ns<br>*p* = ns
**CALd**<br>Flapless grou (Gruop A)<br>Flap group (Gruop B)<br>*p** between groups	2.84 ± 0.70<br>3.39 ± 0.80<br>*p** = ns	2.67 ± 0.57<br>3.19 ± 0.66<br>*p** = ns	2.74 ± 0.56<br>3.08 ± 0.50<br>*p** = ns	2.67 ± 0.59<br>3.02 ± 0.81<br>*p** = ns	*p* = ns<br>*p* = ns
